# Aspartate aminotransferase at large—A case of disappearing macro‐aspartate aminotransferase in a child

**DOI:** 10.1002/jpr3.70275

**Published:** 2026-10-26

**Authors:** Aaron Weston, Megan Rodney, Chris Florkowski, Linda Pike, Chris Sies, Simon Thompson, Richard King, Andrew S. Day

**Affiliations:** ^1^ Paediatrics Department Christchurch Hospital Christchurch New Zealand; ^2^ Canterbury Health Laboratories Christchurch New Zealand; ^3^ Department of Paediatrics and Child Health University of Otago Christchurch Christchurch New Zealand

**Keywords:** child, polyethylene glycol, refrigeration, transaminases

## 1 INTRODUCTION

Aspartate aminotransferase (AST) catalyses the transfer of amino groups from aspartate to alpha‐ketoglutarate, to produce oxaloacetate and glutamate.^1^ Elevations in AST are often associated with hepatocellular injury, but when not associated with alanine aminotransferase (ALT) elevation may also occur through nonhepatic causes.^2^

In some cases, AST can form a complex with an immunoglobulin to create a macroenzyme.^1^ This can result in decreased renal clearance and persistent elevation of serum AST activity, which may create a false impression of liver disease or other pathology in an otherwise healthy person.^2^

Early identification of macro‐AST obviates the need for extensive investigations or assessments in cases where no underlying sinister pathology is present, alleviating not only financial cost but also the burden of anxiety on patients and their families. This report outlines a case of macro‐AST confirmed by polyethylene glycol (PEG) precipitation and provides further support for the observation of declining AST activity on refrigeration as a corroborating feature of macro‐AST.

## 2 CASE REPORT

A 12‐year‐old girl of Mauritian and New Zealand European descent was initially seen in primary care for the assessment of a 6‐month history of nonprogressive intermittent frontotemporal headaches associated with nausea and vomiting. These had occurred monthly, with no associated rash, photophobia or neck stiffness. She was otherwise well, not on regular medications and presented with no focal neurological deficit on examination.

Baseline blood tests completed by her General Practitioner were unremarkable other than an AST of 352 U/L (reference interval 10–50 units/L). This was the first measurement of her liver chemistry. While her headaches resolved over the following weeks, her AST level remained persistently elevated between 352 and 415 U/L on successive measurements. Further investigations were undertaken, including a normal head computed tomography scan, abdominal ultrasound, ceruloplasmin and coeliac serology. Serological evidence of past exposure to Epstein‐Barr virus and cytomegalovirus was present, while hepatitis A and B serology indicated no past infection.

Following assessment in the Paediatric Gastroenterology outpatient clinic 10 months after her first blood test, numerous additional tests were performed. Creatine kinase, α‐fetoprotein, α−1‐antitrypsin phenotype, antinuclear antibodies screen, vitamin B12 and folate, iron studies, lipid screen, serum immunoglobulins, tissue auto‐antibody screen and renal chemistry were all normal. AST was again elevated (421 U/L) with other liver chemistry tests again completely normal (bilirubin 7 µmol/L, ALT 16 U/L, gamma‐glutamyl transferase 12 U/L, albumin 44 g/L). Vitamin D level was insufficient (40 nmol/L), for which she was prescribed replacement therapy. Further monitoring of liver chemistry over the following 6 months showed persistent isolated elevation of AST (between 372 and 428 U/L). AST testing was performed by kinetic ultraviolet test on the Beckman Coulter AU5822 using Beckman Coulter reagents (Beckman Coulter Inc.).

Three months after her initial Paediatric Gastroenterology assessment a further blood sample was collected for PEG precipitation, given concern that this could represent a macroenzyme complex. The AST activity on this occasion was elevated at 372 U/L. This sample was set aside for the PEG precipitation planned to be conducted 1 week later, as it is not a routine test. The AST activity was repeated on the day of planned PEG precipitation to account for any potential minor degradation that may have occured during storage. Repeat measurement on that day (after 1 week of refrigeration), however, showed AST of 51 U/L. Other liver enzymes remained stable over this time, as did the AST on control specimens.

A repeat sample from the patient was urgently processed, with a resultant AST of 428 U/L. The same day, this sample was utilised for the PEG precipitation, alongside two controls. PEG precipitation was conducted manually, where 0.2 mL of the patient's sample was mixed with 0.2 mL of 24% PEG reagent, placed in a 37°C water bath for 20 min, and then once cooled to room temperature centrifuged at 3000 rpm for 5 min. Subsequently 0.2 mL of supernatant was removed and added to 0.2 mL of saline and mixed.

Total AST activity in the patient's PEG‐treated sample was below the assay's limit of quantification (i.e., <12 U/L), representing a post‐PEG recovery of less than 3%, compared with 73% and 77% in two control specimens. In addition, on sample storage at 4°C, AST activity on the patient sample was observed to progressively decline to 39 U/L by 7 days, where it remained stable on further testing on Days 8 and 10 (Figure 1).

**Figure 1:**
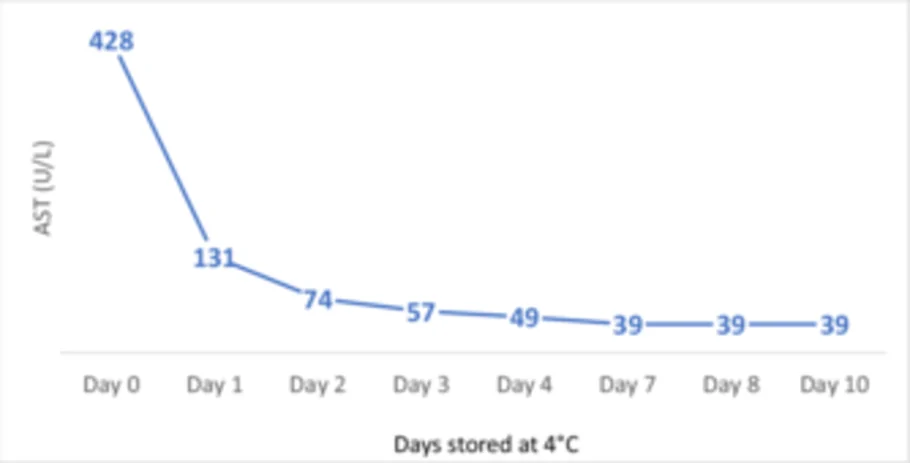
Serial AST results following refrigerated storage of a sample from the patient for 10 days. AST level progressively declined throughout the 10 day period of measurement, reaching a plateau of 39 U/L by Day 7. AST, asparate aminotransferase.

## 3 DISCUSSION

Elevated transaminases are seen in approximately 8% of the general population, with up to 30% of elevations resolving after 3 weeks.^1^ One such transaminase, AST, is present as cytosolic and mitochondrial isoenzymes and is found in a range of cells, including liver, kidneys, pancreas, brain, lungs, cardiac and skeletal muscle, red cells and leucocytes. Cellular injury can trigger the release of AST into the circulation.^1^

With different causes of cellular injury, there is an expected accompaniment of other enzymes. An isolated AST elevation, as seen in macro‐AST, provides a challenge to clinicians and can lead to prolonged investigations and anxiety for children and their families, as was present in the current case.

Estimates of macro‐AST prevalence in children have differed. Caropreso et al.^3^ examined elevated isolated AST in a cohort of 44 children assessed at a tertiary medical centre. Seventeen (38.6%) of these children were shown to have macro‐AST. In contrast, Magen‐Rimon et al.^4^ reported a prevalence as low as 22% in a cohort of 32 children with prolonged isolated AST elevation. Like the majority of the children included in those cohorts, the current case was otherwise healthy.

Various methods have been utilised to detect macro‐AST in plasma. These include PEG precipitation, protein electrophoresis, gel filtration chromatography, measurements of heat stability and precipitation with protein‐A or G‐Sepharose.^5^, ^6^ PEG precipitation can be used to detect macro‐enzymes, as the addition of PEG to the sample leads to precipitation of high molecular weight complexes. After centrifugation, the supernatant can be analysed for enzyme activity, and a post‐PEG recovery can be calculated. In the current case, the presence of macro‐AST was confirmed by a low post‐PEG recovery in comparison to control samples.

Multiple case reports have shown declining AST activity on refrigeration of samples containing macro‐AST when compared to controls.^6^, ^7^, ^8^ Macro‐AST degradation has been postulated as a potentially useful screen in laboratories with limited access to PEG or other complex methodologies.^8^ AST in serum is stable for 4 weeks when stored at 4°C in the absence of macro‐AST, while degradation of AST activity of greater than 90% has been reported after storage at 4°C for 1 week in the presence of macro‐AST.^7^ It has been proposed that the reduced stability of macro‐AST during refrigeration is due to gradual precipitation at cold temperatures, however the underlying mechanism has not been conclusively identified.^6^, ^7^ This was replicated in the current case, where a 90% reduction of AST activity was observed after 1 week of refrigeration, which is supportive of macro‐AST.

## 4 CONCLUSION

This case provides further support that declining AST activity on refrigeration, previously reported in cases with macro‐AST, could act as a corroborating feature and may assist in the identification of macro‐enzymes, especially when PEG studies are not readily available. This case also highlights the entity of macro‐AST as a benign potential cause for isolated elevation of AST in children.

## CONFLICT OF INTEREST STATEMENT

The authors declare no conflicts of interest.

## ETHICS STATEMENT

Informed patient consent was obtained for publication of this case.

## Supporting information

CARE_checklist (1).
